# Human body dynamics simulation and comfort evaluation of interhospital transport patients with different road conditions

**DOI:** 10.1371/journal.pone.0341608

**Published:** 2026-03-17

**Authors:** Min Yao, Xiangjing Kong, Cui Pang, Yizhou Luo, Bao Zhang, Mei Li, Huang Huang, Liguo Zang

**Affiliations:** 1 Eastern Theater Command Air Force Hospital, Nanjing, China; 2 Nanjing Tech University, Nanjing, China,; 3 Nanjing Institute of Technology, Nanjing, China; Federal University of Technology - Parana, BRAZIL

## Abstract

Road unevenness has a significant impact on the comfort of patients during interhospital transport. Vibration is easy to cause dizziness, palpitation, tension, restlessness and other symptoms, and even cause secondary injuries to patients. Therefore, it is very important to analyze the vibration characteristics of human body and take care of vibration reduction. In this paper, the finite element technology, dynamic theory and experimental method are used to establish a “human-vehicle-road” coupling dynamic model, and the human dynamic response under different road conditions is simulated and analyzed. The results show that the human comfort is poor on bumpy road and continuous speed bumps; The vibration of different parts of human body is very different, and the head and sacrococcygeal vibration are the most obvious. Therefore, for patients with interhospital transport, vibration reduction nursing should be focused on the head and sacrococcygeal region. And a variable stiffness damping device is proposed, which has the functions of damping and limiting position. The simulation results show that the vibration reduction of head is more than 35%, and that of sacrococcygeal region is more than 30%. Therefore, the device is of great significance for improving the comfort of patients with interhospital transport.

## 1 Introduction

Interhospital transport is the process of transferring critically ill patients from hospital A to hospital B and ambulance transport is the main way of interhospital transport. As a special vehicle, ambulance plays a role in emergency rescue, patient transport and pre-treatment nursing, which puts forward higher requirements on the comfort of ambulance [1,2]. The key factor affecting the comfort of the ambulance is the road unevenness, and the road unevenness causes the vehicle body to produce medium and low frequency vibration, causing dizziness, palpitation, tension, restlessness and other symptoms of the patient, and even causing secondary injuries to the patient. Especially when the vehicle runs on the bumpy road and over the speed bump, the vibration energy of the body is large, which seriously affects the human comfort [3,4].

The ambulance should not only transport the patient, but also provide a comfortable transport environment for the patient to avoid the deterioration of the patient’s condition. Raine et al. [5] conducted a study on the deterioration of patients’ conditions during transport, and the results showed that about 10% of patients’ conditions deteriorated to varying degrees, and the main reason was that the impact vibration of vehicles directly affected patients and caused secondary damage to their psychological and physiological conditions. At present, the main indexes for evaluating patients’ comfort are vibration acceleration and interface dynamic pressure [6–8]. And the pressure comes from human impact vibration, so the attenuation of human impact vibration is the key to improve patient comfort.

The relationship between objective vibration data and human comfort is specified in GB/T 4970−2009 [9]. The objective data is represented by acceleration a_w_, whose unit is m/s^2^. The corresponding relationship: *a*_*w*_ < 0.315, no uncomfortable (C0); 0.315 ≤ *a*_*w*_ < 0.63, some discomfort (C1); 0.5 ≤ *a*_*w*_ < 1.0, uncomfortable (C2); 0.8 ≤ *a*_*w*_ < 1.6, more uncomfortable (C3); 1.25 ≤ *a*_*w*_ < 2.5, very uncomfortable (C4); *a*_*w*_ > 2.0, extremely uncomfortable (C5). Compared with healthy people, critically ill patients are more sensitive to vibration and require lower vibration levels. For patients in supine position, the main force points of the body are the head, shoulder, elbow, sacrococcygeal region and heel.

**Fig 1:**
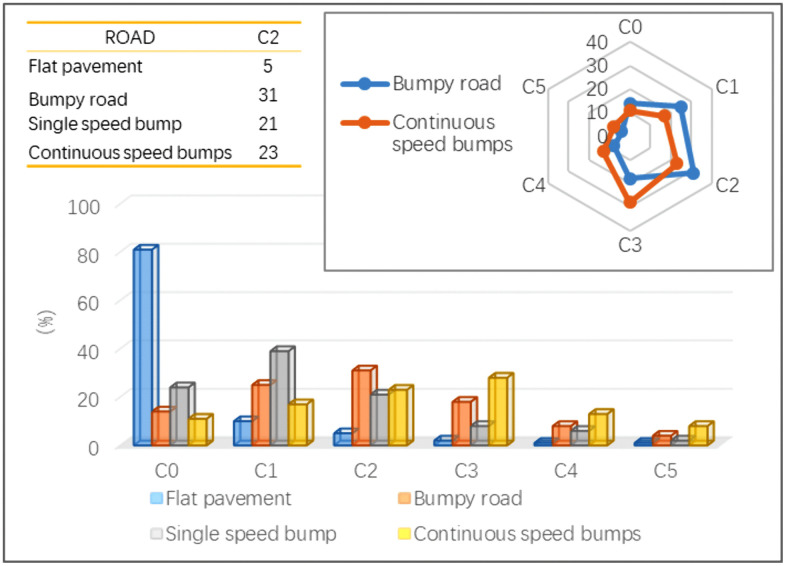
Comfort survey results of different roads.

**Fig 2:**
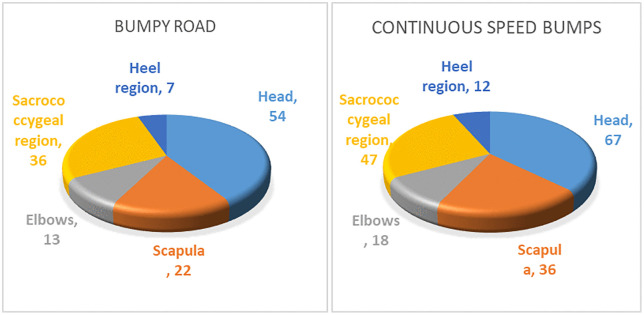
Comfort survey results of different parts of the human body.

The comfort of 100 patients was investigated and the results are shown in Fig 1 and Fig 2. Fig 1 shows the comfort survey results of different road conditions. On flat pavament, there were 81 patients with no discomfort, 10 patients with some discomfort, and 9 patients with the sum of other patients. On bumpy road, the total number of patients with C2, C3, C4 and C5 was 61. Passing single speed bump, the total number of patients with C2, C3, C4 and C5 was 37. Passing continuous speed bumps, the sum of C2, C3, C4 and C5 patients was 72. Therefore, continuous speed bumps and bumpy road have the most obvious influence on patient comfort.

Fig 2 shows the comfort survey results of different parts of the human body. On bumpy road, there were 54 cases of head discomfort, 36 cases of sacrococcygeal discomfort, 22 cases of scapular discomfort, and 20 cases of other parts discomfort. Passing single speed bumps, 67 cases of head discomfort, 47 cases of sacrococcygeal discomfort, 36 cases of scapular discomfort, and 30 cases of other discomfort. Therefore, head and sacrococcygeal vibration has the most obvious influence on patient comfort.

In order to accurately analyze the influence of road roughness on patient comfort, human dynamics studies are usually needed. The main research methods of human dynamics are simulation and experiment. For example, E.Alberti [10] and Ya Huang [11] used experimental methods to analyze the dynamic response of human body in vibration environment, and analyzed the sensitivity of patients to vibration. John Meusch [8] simulated the influence of pavement uneven excitation on human body dynamics through numerical methods, quantified human body vibration level, and analyzed the characteristics of human body vibration distribution. The experimental method can only analyze the local dynamic characteristics of human body. Numerical methods can solve the shortcomings of experimental method, but the existing numerical methods do not consider the influence of nonlinear factors, including material nonlinearity, geometric nonlinearity, boundary nonlinearity and so on. As a result of the above factors, there are still many shortcomings in the existing methods for human body dynamics analysis.

The excitement caused by the road unevenness is transmitted to human body through vehicle and causes human body vibration. Therefore, in order to accurately, comprehensively and intuitively reflect the characteristics and laws of human vibration, it is necessary to comprehensively use simulation and test methods to build a human-vehicle-road coupling model. However, the current research in the field is not deep enough. Based on the current research status, the main research content and innovation points of this paper are as follows:

1. A nonlinear simulation model of human-vehicle-road coupling is created, and factors such as material nonlinearity, geometric nonlinearity and boundary nonlinearity are considered.
2. The dynamic response and vibration distribution of human body are analyzed, and the vibration level of human body is quantified.
3. A damping device with variable stiffness is proposed to realize vibration reduction nursing of key parts of human body.

## 2 Human-vehicle-road coupling dynamic model

### 2.1 Simulation model

**Fig 3:**
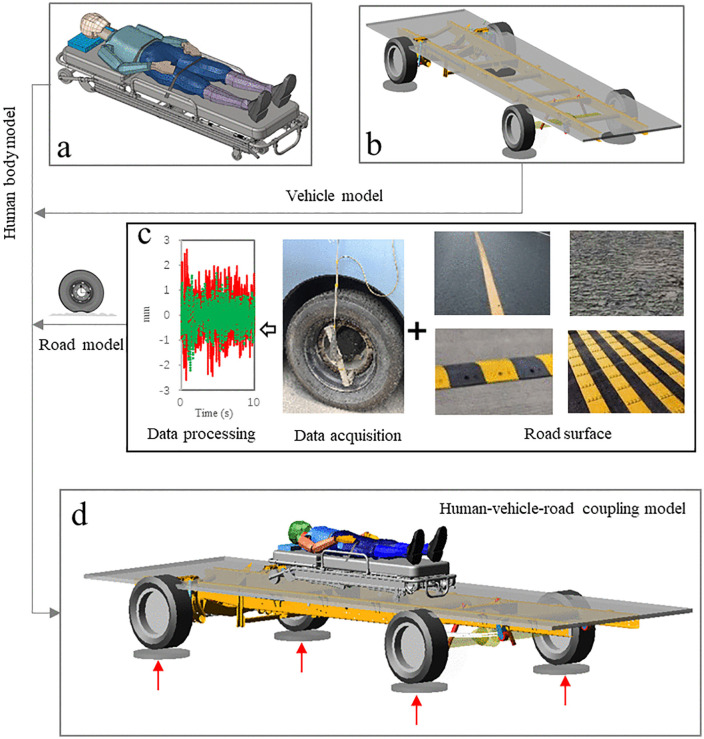
The modeling process of simulation model. a, Human body model. b, Vehicle model. c, Road model. d, Human-vehicle-road coupling model.

In this paper, the finite element method is used to create the human body model, the vehicle model is created according to the dynamics theory, and the road spectrum acquisition technology is used to create the road model [12,13]. The three models are virtual assembled on ADAMS/VEIW platform to create human-vehicle-road coupling model. The modeling process of the simulation model is shown in Fig 3.

The finite element model of human body is shown in Fig 3a. The height of the human body is 1.7 m and the weight is 65 kg. The human body model is composed of skeleton and muscle layer. The skeleton uses solid elements with material density of 1.2 g/cm3, poisson’s ratio of 0.3, and elastic modulus of 3000 MPa. The muscle layer is made of solid elements with material density of 1.06 g/cm3, poisson’s ratio of 0.49, and elastic modulus of 260 MPa. The number of mesh elements is 665,300, with the sizes ranging from 2 to 5 mm. The Jacobi ratio is greater than 0.6, the warpage is less than 5.0 deg, and the aspect ratio is less than 4.0. The human body is in a supine position, and contact units are created between human body and the headrest, stretcher bed, and seat belt. The frame of stretcher bed is made of shell unit and the material is steel, the mattress is made of solid elements with material density of 1.2 g/cm3, poisson’s ratio of 0.4, and elastic modulus of 277 MPa.

The human body joints are connected by spring elements. To ensure the biological fidelity of the human multi-body dynamics model, its joint mechanical parameters are determined through an optimization process based on international standards. Taking the vertical vibration transmission rate of the sitting human body as defined in ISO 5982:2001 as the objective, the stiffness and damping coefficients of the lumbar and cervical vertebrae in the model are optimized parameters to make the overall frequency response function of the model consistent with the standard. The remaining joint parameters are reasonably determined based on the anatomical proportion relationship of the human body and the impedance range described in the Griffin manual. The stiffness of the cervical spring is 2.8 × 10^5^ N·m/rad, and the damping is 120.0 N·m·s/rad. The stiffness of the lumbar spring is 1.6 × 10^5^ N·m/rad, and the damping is 80.0 N·m·s/rad. The spring stiffness of the hip joint is 3.5 × 10^5^ N·m/rad, and the damping is 150.0 N·m·s/rad. The stiffness of the knee joint spring is 9.0 × 10^4^ N·m/rad, and the damping is 50.0 N·m·s/rad. The stiffness of the elbow joint spring is 6.0 × 10^4^ N·m/rad, and the damping is 30.0 N·m·s/rad.

The vehicle dynamics model is shown in Fig 3b. The vehicle model mainly includes front suspension model, rear suspension model, body model and wheel model. The front suspension is a torsion bar spring suspension, and the rear suspension is a leaf spring suspension. The elastic elements and damping elements of the suspension are the key components to attenuate road vibration [14,15]. The stiffness of a torsion bar spring suspension can be expressed as


$$K_{f}=K_{1}(\frac{d\beta }{dx}{)}^{2}+K2(\frac{d\theta }{dx}{)}^{2}+T\frac{d^{2}\beta }{dx^{2}}$$ (1)

Where, *T* stands for torsion bar spring preload, *dβ* stands for angle of the upper control arm, *dθ* stands for angle of the lower control arm, *dx* stands for vertical displacement of the wheel, *K*_*1*_ stands for torsion bar spring stiffness, *K*_*2*_ stands for rubber bushing stiffness.

The stiffness of the leaf spring suspension can be expressed as


$$K_{r}=\frac{P}{f_{r}}$$ (2)

Where, *P* stands for the end load, *fr* stands for spring deflection.

The damper is created using IF function, and the damping expression of the damper is


$$\begin{array}{c} {}*20lF_{d}=IF(v_{ud}+0.4:-967-4987v_{ud},-967,\\ \;\;\;\;\;\;\;IF(v_{ud}+0.2:-589-4030v_{ud},-589,\\ \;\;\;\;\;IF(v_{ud}:-3036v_{ud},0,\\ \;\;\;\;IF(v_{ud}-0.2:19134v_{ud},3754.7,\\ \;\;\;IF\left(v_{ud}-0.4:3754.7+4030v_{ud},4762.9,4762.9+13939v_{ud}\right))))) \end{array}$$ (3)

Where, *v*_*ud*_ stands for speed of the damper.

The rubber cushion block is expressed as


$$F_{r}=0.04{b_{x}}^{4}-1.5113{b_{x}}^{3}+22.14{b_{x}}^{2}-32.511b_{x}+47.878$$ (4)

Where, *b*_*x*_ stands for the compression of the rubber cushion block.

The wheels are created using FTire model with effective frequency range of 0–30 Hz, which meets the requirements of vibration simulation. FTire model is a nonlinear tire model that can identify many different types of road files and has high computational accuracy. The test bench is created under the four wheels respectively, and the wheels are connected to the test benches through contact.

The road model is shown in Fig 3c. Six-direction force sensors are installed at the wheel center position of the four wheels, and the road spectrum data of flat pavament, bumpy road, single speed bump and continuous speed bumps are collected respectively, and the data are cleaned. The displacement time domain data is used as the input of the simulation model.

Modal analysis is performed on the human body finite element model to generate modal neutral files (MNF file). The human MNF file is imported into ADAMS/VIEW platform for virtual assembly with the vehicle model. And the road spectrum data is loaded onto the test bench. Thus, a human-vehicle-road coupled dynamics model is created, as shown in Fig 3d.

### 2.2 Model validation

Dynamics simulation of the human-vehicle-road coupled model is conducted to extract the acceleration data of the human head, sacrococcygeal region, and heels. The simulation data is compared with the experimental data to verify the accuracy of the simulation model. The simulation step size for each operating condition is 1000, the solver is GSTIFF/SI2, and the computational error is 0.01. The simulation data is filtered with a lower cutoff frequency of 0.5 Hz and an upper cutoff frequency of 30 Hz.

**Fig 4:**
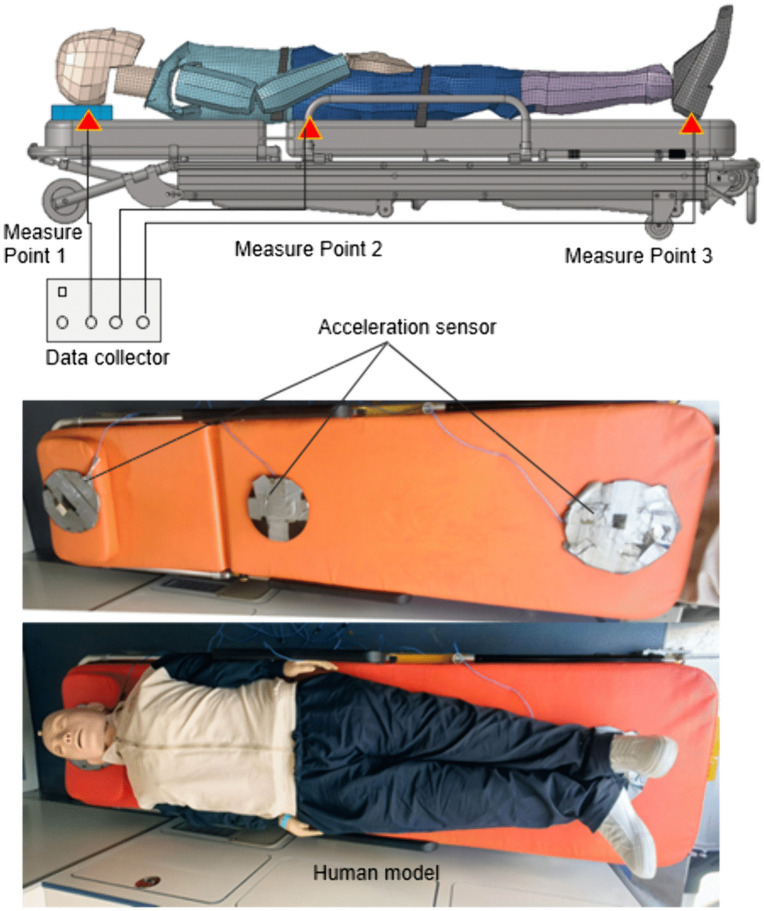
Human body vibration test.

The human body vibration test is shown in Fig 4. The position of test points is consistent with that of simulation data extraction, and the acceleration sensors are placed on the head, sacrococcygeal region, and heels respectively. The acquisition equipment is Siemens LMS, the acquisition frequency range is 0.5–30 Hz, and the resolution is 0.01. Then, the acceleration test data are processed. The human body vibration test and road spectrum acquisition test are carried out simultaneously. Flat pavement and single speed bump are selected for the test, and the human body uses a standard dummy model.

On flat pavement, the maximum and average values of acceleration are compared. For single speed bump, the maximum acceleration is compared. The cross-correlation analysis method [16] was used to compare the trend of data. The cross-correlation analysis between two data sets *y*_*1*_*(t)* and *x*_*1*_*(t)* can be expressed as


$$\begin{array}{c} \begin{array}{c} R_{xy}(\tau )=E\left[y(t)x(t-\tau )\right]\\ \;\;\;=E\left\{\left[y_{1}(t)+\nu_{2}(t)\right]\left[x_{1}(t-\tau )+\nu_{1}(t-\tau )\right]\right\}\\ \;\;\;=E\{y_{1}(t)x_{1}(t-\tau )+y_{1}(t)\nu_{1}(t-\tau )+\\ \;\;\;\nu_{2}(t)x_{1}(t-\tau )+\nu_{2}(t)\nu_{1}(t-\tau )\}\\ \;\;\;=E\left[y_{1}(t)x_{1}(t-\tau )\right]+E\left[y_{1}(t)\nu_{1}(t-\tau )\right]+\\ \;\;\;E\left[\nu_{2}(t)x_{1}(t-\tau )\right]+E\left[\nu_{2}(t)\nu_{1}(t-\tau )\right]\\ \;\;\;=R_{y_{1}x_{1}}(\tau )+R_{y_{1}\nu_{1}}(\tau )+R_{\nu_{2}x_{1}}(\tau )+R_{\nu_{2}\nu_{1}}(\tau )=R_{y_{1}x_{1}}(\tau ) \end{array} \end{array}$$ (5)

Where, *ν*_*1*_*(t)* and *ν*_*2*_*(t)* are interference signals, τ stands for time delay, and $R_{y_{1}\nu_{1}}(\tau )=R_{\nu_{2}x_{1}}(\tau )=R_{\nu_{2}\nu_{1}}(\tau )=0$.

**Fig 5:**
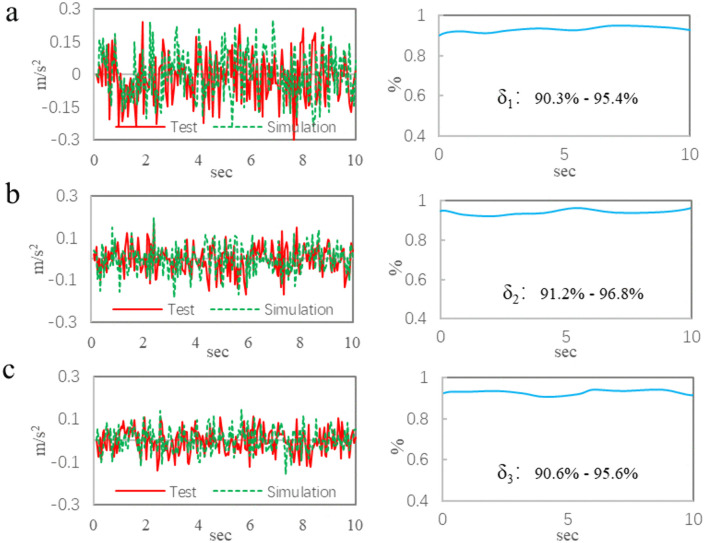
The comparison results on uneven road. a, Data of the point 1. b, Data of the point 2. c, Data of the point 3.

Fig 5 shows the comparison results on uneven road, and the comparison data for test point 1 are shown in Fig 5a. The test data of the maximum acceleration is 0.28 m/s^2^, the simulation data is 0.26 m/s^2^, and the agreement is 92.3%; The test data of the average acceleration is 0.191 m/s^2^, the simulation data is 0.172 m/s^2^, and the agreement is 90.1%; The trends of the two curves are consistent, with the correlation coefficient ranging from 90.3% to 95.4%.

The comparison data for test point 2 are shown in Fig 5b. The maximum acceleration of test value is 0.25 m/s^2^, and that of the simulation value is 0.23 m/s^2^, and the agreement is 92.0%; The average acceleration of test data is 0.185 m/s^2^, the simulation data is 0.167 m/s^2^, and the agreement is 90.3%; The correlation coefficients between the two curves range from 91.2% to 96.8%.

Fig 5c shows the comparison results of test point 3. The maximum acceleration of test value is 0.24 m/s^2^, the simulation value is 0.22 m/s^2^, and the agreement is 91.7%; The average acceleration of test data is 0.173 m/s^2^, the simulation data is 0.161 m/s^2^, and the agreement is 93.1%; The correlation coefficients of the two curves range from 90.6% to 95.6%.

**Fig 6:**
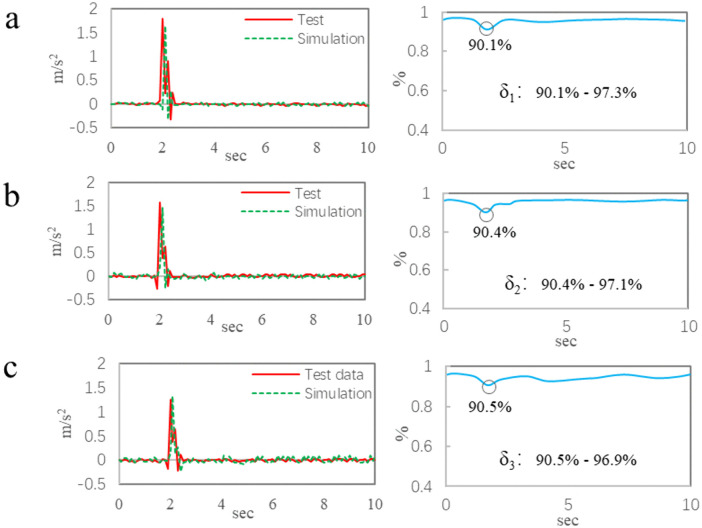
The comparison results of single speed bump. a, Data of the point 1. b, Data of the point 2. c, Data of the point 3.

Fig 6 shows the comparison results of single speed bump. In Fig 6a, the maximum acceleration of test value at the point 1 is 1.78 m/s^2^, the simulation value is 1.65 m/s^2^, and the agreement is 92.7%; The correlation coefficients of the two curves range from 90.1% to 97.3%. The comparison data for the point 2 are shown in Fig 6b, the maximum acceleration of test value is 1.58 m/s^2^, and that of the simulation value is 1.46 m/s^2^, and the agreement is 92.5%; The correlation coefficients between the two curves range from 90.4% to 97.1%. In Fig 6c, the maximum acceleration of test value at the point 3 is 1.16 m/s^2^, the simulation value is 1.23 m/s^2^, and the agreement is 94.3%; The correlation coefficients of the two curves range from 90.5% to 96.9%.

**Fig 7:**
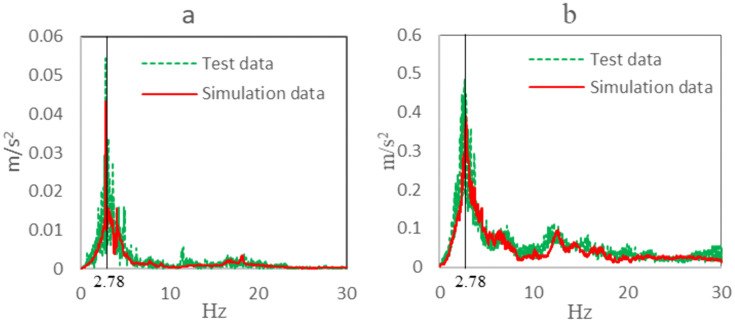
Comparison of frequency domain data. a, Uneven road. b, Single speed bump.

Taking measurement point 2 as an example, the agreement between simulation and test data in the frequency domain is compared. Fig 7a shows the frequency-domain data on the uneven road. The peak frequency of the test data is 2.78 Hz and the amplitude is 0.056 m/s ²; The peak frequency of the simulation data is 2.69 Hz and the amplitude is 0.048 m/s ². The natural frequency coincidence degree is 96.8%, and the amplitude coincidence degree is 85.7%.

Fig 7b shows the frequency-domain data of a single speed bump. The peak frequency of the test data is 2.78 Hz and the amplitude is0.49 m/s ². The peak frequency of the simulation data is 2.73 Hz and the amplitude is 0.43 m/s ². The coincidence degree of the natural frequencies of the two is 98.2%, and the coincidence degree of the amplitudes of the two is 87.8%.

In time domain, the coincidence degrees of the two data are both greater than 90%. In frequency domain, the coincidence degrees of the two data are both greater than 90%. The comparison results show that the dynamic simulation model established in this paper is accurate, and the simulation results can truly reflect the dynamic characteristics of the human body.

## 3 Comfort analysis

**Fig 8:**
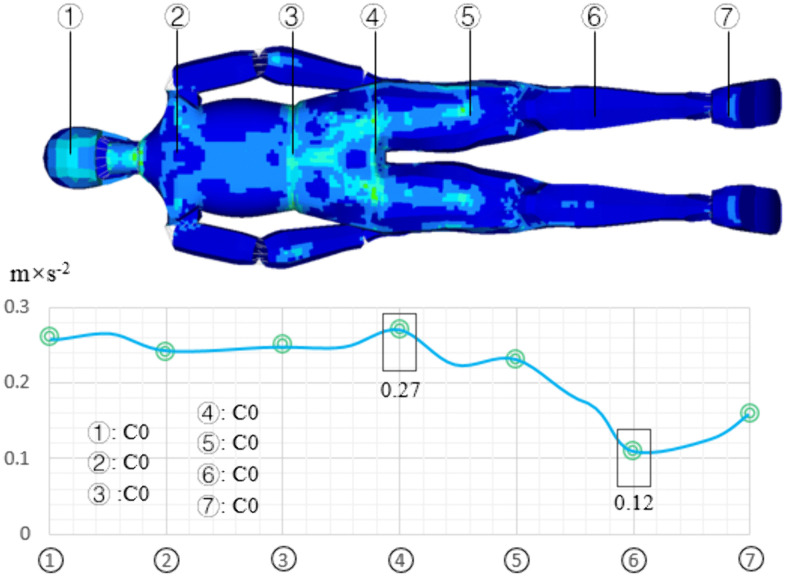
The vibration distribution of human body on flat pavement.

The vibration distribution of human body on flat pavement is shown in Fig 8. The vibration in the sacrococcygeal region is the highest, with a value of 0.27 m/s^2^, the vibration of lower leg is the smallest, and its value is 0.12 m/s^2^, the head vibration acceleration is 0.26 m/s^2^. The distribution of human body vibration indicates that on flat pavement, the main areas of human body vibration are the sacrococcygeal region and head, with less vibration in other areas. The human body vibration acceleration is less than 0.3 m/s^2^, and the comfort evaluation result is C0 (no uncomfortable).

**Fig 9:**
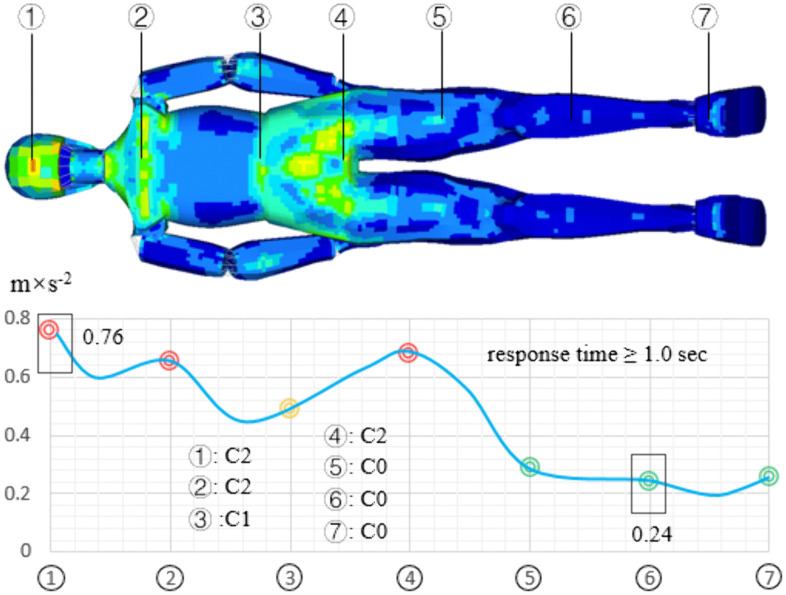
The vibration distribution of human body on bumpy road.

The vibration distribution of human body on bumpy road is shown in Fig 9. The vibration in head is the highest, with a value of 0.76 m/s^2^, the vibration of lower leg is the smallest, and its value is 0.24 m/s^2^, the sacrococcygeal region vibration acceleration is 0.68 m/s^2^. The vibration of human body mainly distributed in the head, shoulder and sacrococcygeal region, and the vibration of the lower leg and heel is small. The comfort evaluation results for the head, shoulder, and sacrococcygeal region are C2 (uncomfortable); The comfort evaluation result for the waist is C1 (some discomfort); the comfort evaluation result for the lower leg and heel is C0 (no uncomfortable). On this road, the human body response time is much greater than 1 sec, the subjective feeling of human comfort becomes worse after a long time of large vibration. Therefore, on bumpy road, the subjective evaluation of comfort is worse than the theoretical evaluation of comfort.

**Fig 10:**
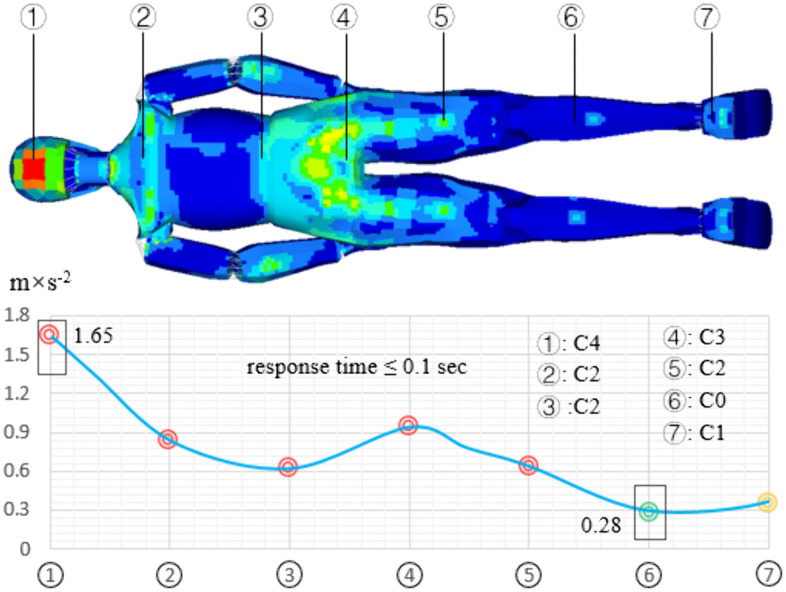
The distribution of human body vibration passing a single speed bump.

The distribution of human body vibration passing a single speed bump is shown in Fig 10. The vibration in the head is the largest, with a value of 1.65 m/s^2^; the vibration in the lower leg is smallest, with a value of 0.28 m/s^2^; the vibration acceleration in the sacrococcygeal region is 0.94 m/s^2^. For a single speed bump, human vibration is mainly distributed in the head and the sacrococcygeal region. The comfort evaluation result for the head is C4 (very uncomfortable); the result of the sacrococcygeal region is C3 (more uncomfortable); the results for the shoulder, waist, and thigh are C2 (uncomfortable); The comfort evaluation results for the lower leg and heel are C0 (no uncomfortable). For this pavement, the human body response time is less than 0.1 sec. Therefore, the subjective evaluation of comfort is better than the theoretical evaluation result.

**Fig 11:**
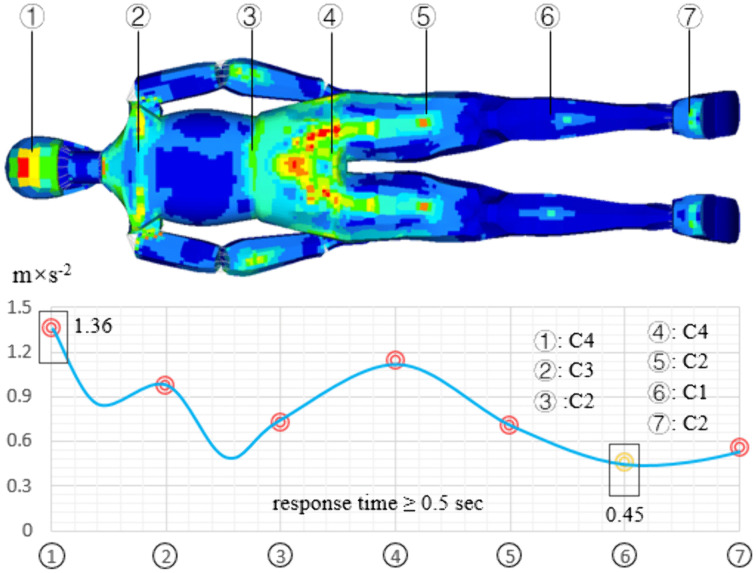
The distribution of human vibration passing continuous speed bumps.

Fig 11 shows the distribution of human body vibration on consecutive speed bumps. The maximum acceleration occurs at the head, and its value is 1.36 m/s^2^; the minimum acceleration occurred in the lower leg, which is 0.45 m/s^2^; the sacrococcygeal vibration acceleration is 1.14 m/s^2^. For continuous speed bump, human vibration is mainly distributed in the head and the sacrococcygeal region. The comfort evaluation result for the head is C4 (very uncomfortable); the result for the sacrococcygeal region is C3 (more uncomfortable); the result for the shoulder, waist, and thigh is C2 (uncomfortable); the result for the lower and heet is C1 (some uncomfortable). On the pavement, the human response time is greater than 0.5 sec, and continuous high-intensity vibration reduces human comfort. Therefore, the subjective evaluation of comfort is worse than the theoretical evaluation of comfort.

The results show that the vibration of head and sacrococcygeal region is the most obvious and has the greatest influence on human comfort. Therefore, during inter-hospital transfer, it is important to focus on vibration reduction nursing for patient’s head and sacrococcygeal region to improve the patient’s comfort.

## 4 Vibration reduction nursing

At present, there are two ways to improve the comfort of ambulance: one is the optimal design of vehicle suspension system, and the other is the comfort design of ambulance stretcher bed. In terms of reducing road excitation transmission, the most effective measure is to improve suspension vibration isolation rate through optimization [17,18]. However, the vibration isolation rate of most ambulance suspensions has reached the level of 85%−90%, considering the stability and reliability of the vehicle, the improvement of suspension vibration isolation rate has been very limited. Therefore, it is very important to study the damping performance of ambulance stretcher bed.

At present, most ambulance stretcher beds are directly fixed on the body floor, and the body vibration is directly transmitted to the patient. In order to reduce vibration transmission, it is necessary to install a set of elastic devices between the body floor and the stretcher bed. The most common elastic device is composed of springs and shock absorbers [19]. In order to achieve an ideal vibration reduction effect, the stiffness of the spring is often designed to be small, but this also increases the dynamic stroke and instability of the stretcher bed, which also brings umcomfort and insecurity to patients. Therefore, the damping performance and stability performance of the elastic device of the ambulance stretcher bed are equally important.

**Fig 12:**
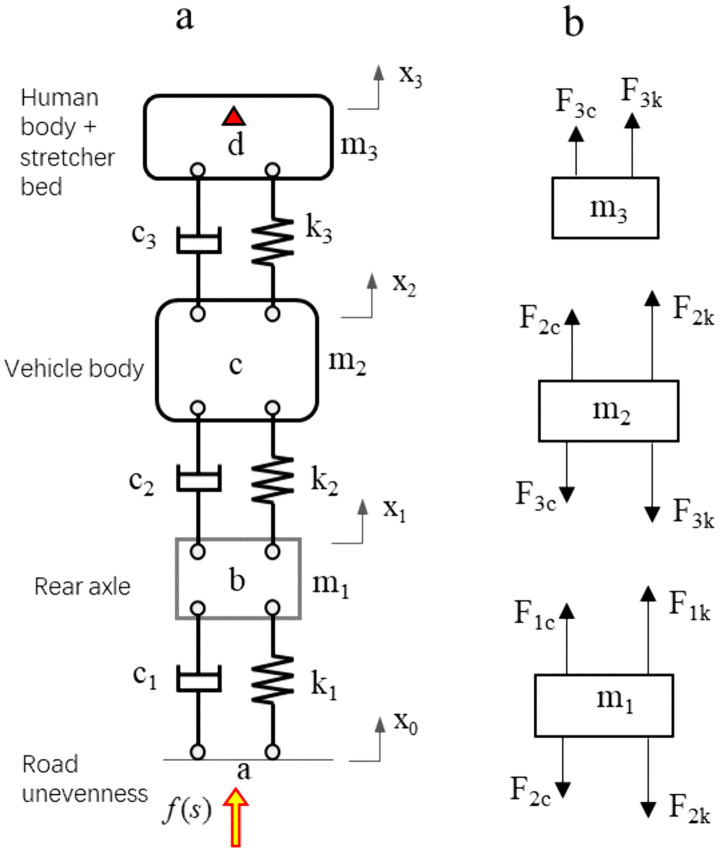
The System dynamics model. a, Vibration transmission model. b, Force model.


In order to reduce human body vibration, a set of air bomb vibration damping devices is designed between the stretcher bed and the vehicle body floor. The road unevenness is transmitted to the stretcher bed and the human body through the path of tire – suspension – air spring vibration damping device. The equivalent dynamic model is shown in Fig 12. And Fig 12a stands for vibration transmission model, Fig 12b stands for force model. Taking (*x*_*1*_*, x*_*2*_*, x*_*3*_) as the generalized coordinates, the vibration equation of the system is established as

$$\left\{ \begin{array}{c} @lm_{\text{1}}\overset{\cdot \cdot }{x_{\text{1}}}-(F_{1k}+F_{1c})+(F_{2k}+F_{2c})=0\\ m_{\text{2}}\overset{\cdot \cdot }{x_{\text{2}}}-(F_{2k}+F_{2c})+(F_{3k}+F_{3c})=0\\ m_{\text{3}}\overset{\cdot \cdot }{x_{\text{3}}}-F_{3k}-F_{3c}=0 \end{array}\right.$$ (6)

According to Eq. (3), there is


$$\begin{array}{c} \left\{ \begin{array}{c} @lm_{1}\overset{⬝⬝}{x_{1}}\;-c_{1}(\overset{⬝}{x_{1}}\;-\overset{⬝}{x_{0}}\;)+c_{2}(\overset{⬝}{x_{2}}\;-\overset{⬝}{x_{1}}\;)-k_{1}(x_{1}-x_{0})+k_{2}(x_{2}-x_{1})=0\\ m_{2}\overset{⬝⬝}{x_{2}}\;-c_{2}(\overset{⬝}{x_{2}}\;-\overset{⬝}{x_{1}}\;)+c_{3}(\overset{⬝}{x_{3}}\;-\overset{⬝}{x_{2}}\;)-k_{2}(x_{2}-x_{1})+k_{3}(x_{3}-x_{2})=0\\ m_{3}\overset{⬝⬝}{x_{3}}\;-c_{3}(\overset{⬝}{x_{3}}\;-\overset{⬝}{x_{2}}\;)-k_{3}(x_{3}-x_{2})=0 \end{array}\right. \end{array}$$ (7)

Where, *k*_*1*_ stands for tire stiffness, *k*_*2*_ stands for suspension stiffness, *k*_*3*_ stands for air spring stiffness, *c*_*1*_ stands for tire damping, *c*_*2*_ stands for suspension damping, *c*_*3*_ stands for air spring damping, *m*_*1*_ stands for the axle mass, *m*_*2*_ stands for the vehicle body mass, and *m*_*3*_ stands for the stretcher bed and human body mass.

The Laplace transform of Eq. (4) is


$$\left\{ \begin{array}{c} @lm_{1}s^{2}x_{1}(s)+(c_{2}s+k_{2})\left[x_{2}(s)-x_{1}(s)\right]-(c_{1}s+k_{1})\left[x_{1}(s)-x_{0}(s)\right]=0\\ m_{2}s^{2}x_{2}(s)-(c_{2}s+k_{2})\left[x_{2}(s)-x_{1f}(s)\right]+(c_{3}s+k_{3})\left[x_{3}(s)-x_{2}(s)\right]=0\\ m_{3}s^{2}x_{3}(s)-(c_{3}s+k_{3})\left[x_{3}(s)-x_{2}(s)\right]=0 \end{array}\right.$$ (8)

The transfer function of the sub-paths are


$$H_{ab}(s)=\frac{x_{1}(s)}{x_{0}(s)},H_{bc}(s)=\frac{x_{2}(s)}{x_{1}(s)},H_{cd}(s)=\frac{x_{3}(s)}{x_{2}(s)}$$ (9)

Then, the transfer function of the total transfer path is


$$H_{abcd}(s)=H_{ab}(s)\cdot H_{bc}(s)\cdot H_{cd}(s)$$ (10)

By reducing the transfer function Habcd(S), the influence of road unevenness on human vibration can be effectively reduced. According to Eq. (8), Eq. (9) and Eq. (10), Habcd(S) can be reduced by optimizing *k*_*3*_ and *c*_*3*_. The sensitive frequency of human body is 4 Hz – 6 Hz, and the natural frequency of the suspension system is 1.5 Hz – 2.2 Hz. Therefore, the constraint equation is


$$f_{3}=\frac{\text{1}}{2\pi }\sqrt{\frac{k_{3}}{m_{3}}},3.0Hz\leq f_{3}\leq 3.5Hz$$ (11)

According to Eq. (11), the initial stiffness of k_3_ is 34 N/mm. And the initial damping coefficient of c_3_ is 0.6. The parameters of the system dynamics model are shown in Table 1.

**Table 1:** Parameters of the system.

Stiffness (N.mm^-1^)	k_1_ = 1400, k_2_ = 290, k_3_ *= 34*
Damping (N.s.mm^-1^)	*c*_*1*_ *= 0.7, c*_*2*_ *= 0.5, c*_*3*_ *= 0.6*
Mass (kg)	*m*_*1*_ *= 215, m*_*2*_ *= 3400, m*_*3*_ *= 150*

The optimization model is


$$\begin{array}{c} \min(\overset{\cdot \cdot }{x_{3}})=f(k_{3},c_{3})\\ 34N/mm\times 80\%\leq k_{3}\leq 34N/mm\times 120\%,\\ 0.6\times 80\%\leq c_{3}\leq 20.6\times 120\%,\\ x\leq 20mm \end{array}$$ (12)

The optimization results of the stiffness and damping of a air spring are 36.5 N/mm and 0.73.

**Fig 13:**
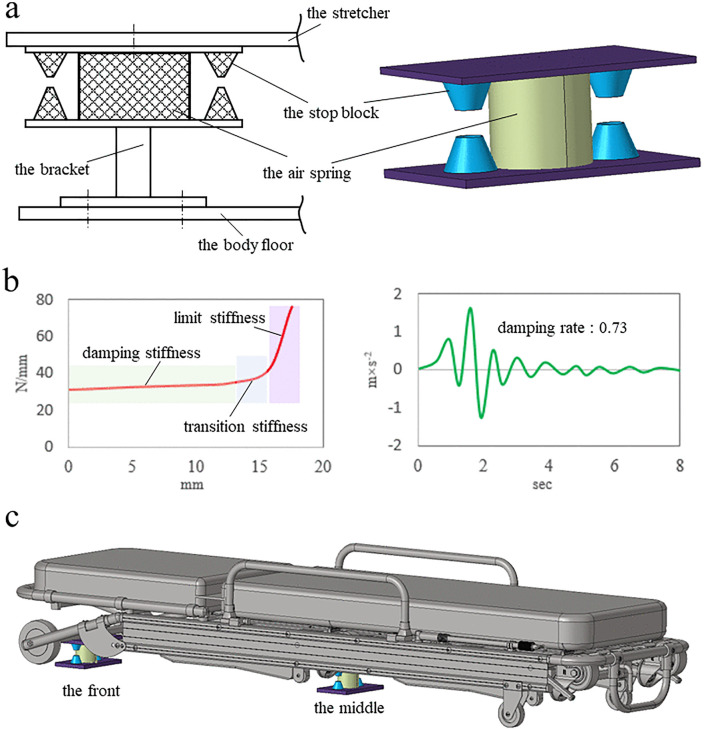
Vibration damping device of a stretcher bed. a, Structure of the device. b, Parameters of the device. c, Arrangement position of the device.

Fig 13a shows a variable stiffness air spring elastic device, which is composed of a bracket, an air spring and two stop blocks, and is installed between the stretcher bed and the body floor. The height of the bracket can be adjusted according to the structure of the stretcher bed, and the material of the stop block is rubber. Fig 13b shows the stiffness curve and damping curve of the device. The stiffness curve includes damping stiffness, transition stiffness and limiting stiffness. The damping curve reflects vibration attenuation level of the device, and the rate of the device is 73%. Fig 13c shows the installation positions of the vibration damping devices, which are located at the front and middle of the stretcher bed.

**Fig 14:**
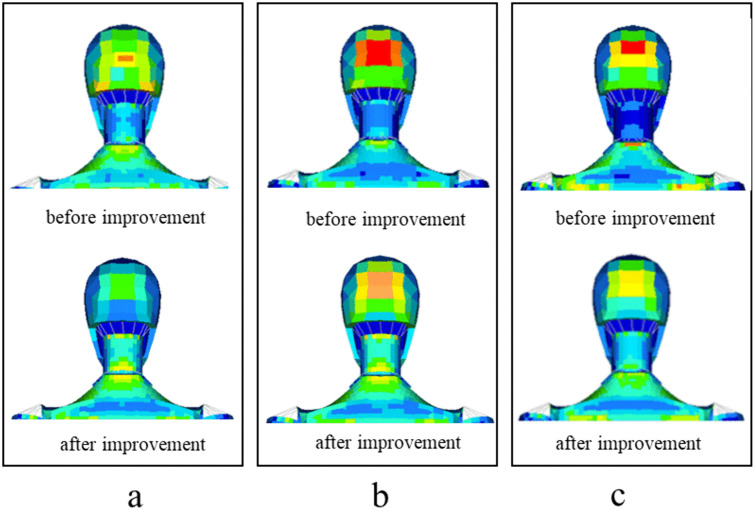
Comparison of head vibration.

Fig 14 shows the comparison results of head vibration before and after using the elastic device. The comparison results of bumpy pavement are shown in Fig 14a. The acceleration before improvement is 0.76 m/s^2^, the acceleration after improvement is 0.49 m/s^2^, and the vibration reduction is 35.5%. The evaluation result of comfort changed from C2 to C1.

Fig 14b shows the comparison results of a single speed bump. The acceleration before improvement is 1.65 m/s^2^, the acceleration after improvement is 0.96 m/s^2^, and the vibration reduction is 41.8%. The result of comfort evaluation changed from C4 to C3.

Fig 14c shows the comparison results of continuous speed bumps. The acceleration before improvement is 1.36 m/s^2^, the acceleration after improvement is 0.71 m/s^2^, and the vibration reduction is 47.8%. The result of comfort evaluation changed from C4 to C2.

**Fig 15:**
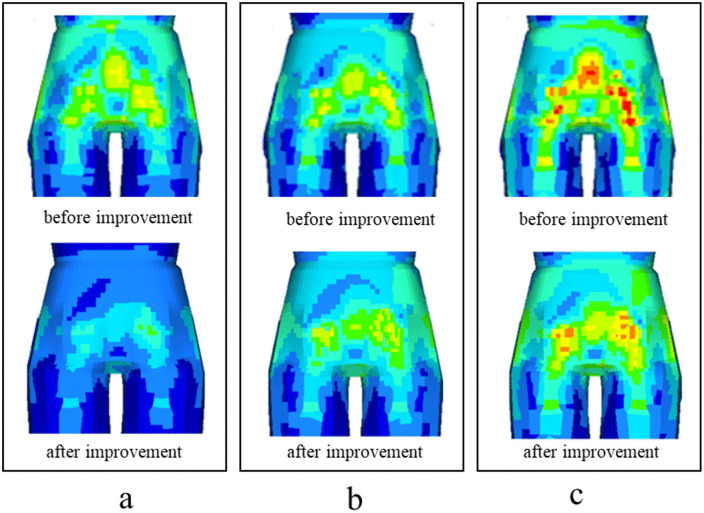
Comparison of sacrococcygeal region vibration.

Fig 15 shows the comparison results of sacrococcygeal region vibration before and after using the elastic device. The comparison results of bumpy pavement are shown in Fig 15a. The acceleration before improvement is 0.68 m/s^2^, the acceleration after improvement is 0.43 m/s^2^, and the vibration reduction is 36.8%. The evaluation result of comfort changed from C2 to C1.

Fig 15b shows the comparison results of a single speed bump. The acceleration before improvement is 0.94 m/s^2^, the acceleration after improvement is 0.62 m/s^2^, and the vibration reduction is 34.1%. The result of comfort evaluation changed from C3 to C2.

Fig 15c shows the comparison results of continuous speed bumps. The acceleration before improvement is 1.14 m/s^2^, the acceleration after improvement is 0.78 m/s^2^, and the vibration reduction is 31.6%. The result of comfort evaluation changed from C3 to C2.

The comparison results show that the device proposed in this paper can effectively reduce the head and sacrococcygeal vibration, which is conducive to improving the comfort of patients with interhospital transport.

## 5 Conclusion

In this paper, according to finite element technology, dynamic theory and test method, the human-vehicle-road coupling model is created. The agreement between the simulation data and the test data is more than 90%. The comparison results show that the model is accurate, and the simulation results can truly reflect the dynamic characteristics of human body. On this basis, the vibration characteristics of human body are simulated and analyzed. The simulation results show that bumpy pavement and continuous speed bumps have the greatest influence on human comfort. There are great differences in the vibration of different parts of human body, among which the vibration of the head and sacrococcygeal region is the most obvious, which are also the key parts affecting the comfort of human body. Therefore, during inter-hospital transfer, it is important to focus on vibration reduction nursing for patient’s head and sacrococcygeal region to improve the patient’s comfort.

In order to improve the patient’s comfort during inter-hospital transfer, a variable stiffness elastic device is proposed in this paper, which consists of an air spring and two rubber stop blocks. The device has both vibration reduction and limiting functions, which not only enhance comfort but also improve stability. The simulation results show that the vibration reduction of the head is more than 35%, and that of the sacrococcygeal region is more than 30% by using the elastic device.

## Supporting information

S1 File Data. (DOCX)
