# Supplementary material for: Human body dynamics simulation and comfort evaluation of interhospital transport patients with different road conditions
Source: PLoS One. 2026 Mar 17;21(3):e0341608. doi: 10.1371/journal.pone.0341608 (PMC12994836; doi:10.1371/journal.pone.0341608)
Supplement: S1 File — (DOCX) [file pone.0341608.s001.docx]

| **Fig. 1** | C0 | C1 | C2 | C3 | C4 | C5 |
| --- | --- | --- | --- | --- | --- | --- |
| Flat pavement | 81 | 10 | 5 | 2 | 1 | 1 |
| Bumpy road | 14 | 25 | 31 | 18 | 8 | 4 |
| Single speed bump | 24 | 39 | 21 | 8 | 6 | 2 |
| Continuous speed bumps | 11 | 17 | 23 | 28 | 13 | 8 |

| **Fig. 2** |  |  |  |  |  |
| --- | --- | --- | --- | --- | --- |
| Bumpy road | Head | Scapula | Elbows | Sacrococcygeal region | Heel region |
|  | 54 | 22 | 13 | 36 | 7 |
| Continuous speed bumps | Head | Scapula | Elbows | Sacrococcygeal region | Heel region |
|  | 67 | 36 | 18 | 47 | 12 |

| **Fig. 8** | Body part | 1 | 2 | 3 | 4 | 5 | 6 | 7 |
| --- | --- | --- | --- | --- | --- | --- | --- | --- |
|  | Test data （m/s^2^） | 0.26 | 0.24 | 0.25 | 0.27 | 0.23 | 0.12 | 0.16 |
|  |  |  |  |  |  |  |  |  |
| **Fig. 9** | Body part | 1 | 2 | 3 | 4 | 5 | 6 | 7 |
|  | Test data （m/s^2^） | 0.76 | 0.65 | 0.49 | 0.68 | 0.29 | 0.24 | 0.26 |
|  |  |  |  |  |  |  |  |  |
| **Fig. 10** | Body part | 1 | 2 | 3 | 4 | 5 | 6 | 7 |
|  | Test data （m/s^2^） | 1.65 | 0.83 | 0.62 | 0.94 | 0.63 | 0.28 | 0.35 |
|  |  |  |  |  |  |  |  |  |
| **Fig. 11** | Body part | 1 | 2 | 3 | 4 | 5 | 6 | 7 |
|  | Test data （m/s^2^） | 1.36 | 0.97 | 0.73 | 1.14 | 0.71 | 0.45 | 0.56 |
